# The Committee Stage of the National Insurance Bill

**Published:** 1911-07-08

**Authors:** 


### THE COMMITTEE STAGE OF THE NATIONAL INSURANCE BILL.

The Committee stage of the National Insurance Bill was begun on Wednesday, July 5, and the Government hopes to secure the passage of the measure by the ordinary facilities under the rules of the House; it is understood that the eleven o'clock rule will be suspended throughout the debate on the remaining stages of the Bill, and it is now thought likely that the session will close about the third week in September. A Parliamentary paper has been issued containing replies to letters addressed to the Chancellor of the Exchequer dealing with various features of the Bill. Two of these replies relate to the position of hospitals under the proposed scheme; in one of them the Chancellor stated that there is no reason why "the utility of a cottage hospital should be interfered with . . . the societies will have power to subscribe to it in the same way as they do now, and will presumably wish to use it for some of their members." In the other letter the Chancellor said he did not think the proposals of the Bill would diminish the need for or the utility of hospitals, and he pointed out that "the experience of Germany, the only other country which has adopted a scheme of compulsory national insurance, shows that the number of public and private hospitals has greatly increased by reason of such insurance." The purport of most of the replies to letters relating to medical service under the proposed scheme has already been stated in The Hospital.

The list of proposed amendments is still increasing, but few that have been added since last week embody new proposals, many of them being in similar terms to those of which notice had already been given. For instance, the amendment providing that provision for medical attendance shall be made by local health committees only, has been put down in the names of Mr. Gibbs, Dr. Addison, Sir Philip Magnus, Dr. Hillier, and the Earl of Ronaldshay. A notice of motion appearing on the paper was in the name of Sir Henry Craik, whose intention it was to move, on the order for Committee on the Bill being read, "that it be an instruction to the Committee on the Bill that they have power to postpone the provisions for medical benefits so that they may be made the subject of a separate Bill." Interviews have recently taken place between the Chancellor and representatives of the British Medical Association, and it is understood that the Chancellor has agreed to the principles of the "free choice of doctor" and medical representation in administration. He is not, however, prepared to reduce the income limit of married persons, and any proposal to effect such a reduction as that suggested by the British Medical Association is likely to meet with strong opposition from Labour members as well as from other sections of the House.

Among the numerous questions that have been asked in the House of Commons relative to the proposed scheme were several of medical interest. Sir Clement Hill asked whether the Chancellor was aware of the existence of provident dispensaries which are charitably aided and secure to their members and their families, as well as to their widows and children, complete medical attention and medicine for sums varying from one penny to threepence a week; and whether he had considered the injurious effect which the compulsory payments under the National Insurance Bill were likely to have upon such organisations. Mr. Lloyd George replied that it was the intention of the Bill that existing organisations which wholly or partly cover the ground to be included should be preserved and uilised rather than displaced. Mr. Astor asked whether a woman confined in a hospital or other institution would receive maternity benefit under the Bill and, if so, would any portion of it be given to the institution. The reply to the first part of the question was in the negative, and therefore the second part does not arise; the Chancellor, however, is in communication on this matter with persons responsible for the management of homes which receive maternity cases. An interesting reply of the Chancellor's was that in which he stated, in answer to a question by Mr. Cassel, that the average number of days' sickness recorded for persons under the German Sickness Insurance Law in 1908 (the latest year for which complete returns have been published) was eight and a half. A number of questions had special reference to sanatorium treatment, but few of them raised points that have not already been dealt with. Major Anstruther-Gray asked whether the Chancellor's attention had been drawn to the treatment of phthisis by radio-active menthol iodine, and whether before embarking on the expense of sanatoria he would carefully consider alternative methods of combating the scourge. In reply Mr. Lloyd George pointed out that the treatment referred to is still in an experimental stage, but that the Bill empowers the Insurance Commissioners to expend upon research a part of the additional State grant for sanatorium benefit. It appears that associations of "medical herbalists" have sent communications to the Chancellor requesting that facilities shall be given to those who prefer to undergo herbalist treatment, and the Chancellor is considering these communications.

